# Clinical, Cytogenetic, and Biochemical Analyses of a Family with a t(3;13)(q26.2;p11.2): Further Delineation of 3q Duplication Syndrome

**DOI:** 10.1155/2013/895259

**Published:** 2013-09-18

**Authors:** M. Abreu-González, C. García-Delgado, A. Cervantes, A. Aparicio-Onofre, R. Guevara-Yáñez, R. Sánchez-Urbina, M. P. Gallegos-Arreola, A. Luna-Angulo, F. J. Estrada, V. F. Morán-Barroso

**Affiliations:** ^1^Department of Genetics, Hospital Infantil de México Federico Gómez, Calle Dr. Márquez 162, Colonia Doctores Del. Cuauhtémoc, 06720 Mexico City, DF, Mexico; ^2^Genetics Service, Hospital General de México Dr. Eduardo Liceaga, Calle Dr. Balmis 148, 06726 Mexico City, DF, Mexico; ^3^Medicine Faculty, UNAM, Avenida Universidad 3000, 04510 Mexico City, DF, Mexico; ^4^Biogen Laboratory, Calle Dr. Vertiz 247-A, 06720 Mexico City, DF, Mexico; ^5^Molecular Medicine Division, CIBO, IMSS, Calle Sierra Mojada 800, 44340 Guadalajara, JAL, Mexico; ^6^Molecular Biology Laboratory, Science Health Faculty, Universidad Panamericana, Calle Donatello 59, 03920 Mexico City, DF, Mexico

## Abstract

Chromosomal abnormalities that result in genomic imbalances are a major cause of congenital and developmental anomalies. Partial duplication of chromosome 3q syndrome is a well-described condition, and the phenotypic manifestations include a characteristic facies, microcephaly, hirsutism, synophrys, broad nasal bridge, congenital heart disease, genitourinary disorders, and mental retardation. Approximately 60%–75% of cases are derived from a balanced translocation. We describe a family with a pure typical partial trisomy 3q syndrome derived from a maternal balanced translocation t(3;13)(q26.2;p11.2). As the chromosomal rearrangement involves the short arm of an acrocentric chromosome, the phenotype corresponds to a pure trisomy 3q26.2-qter syndrome. There are 4 affected individuals and several carriers among three generations. The report of this family is relevant because there are few cases of pure duplication 3q syndrome reported, and the cases described here contribute to define the phenotype associated with the syndrome. Furthermore, we confirmed that the survival until adulthood is possible. This report also identified the presence of glycosaminoglycans in urine in this family, not related to the chromosomal abnormality or the phenotype.

## 1. Introduction

Chromosomal abnormalities that result in genomic imbalances are a major cause of congenital and developmental anomalies including multiple congenital malformations and mental retardation [1]. They can result from a numerical imbalance or from a structural change in the chromosomes resulting in partial deletions and/or duplications. Partial duplication of chromosome 3q syndrome (dup(3q)) is a well-described condition that has overlapping features with the Brachmann de Lange syndrome (MIM 122470) [2–4]. The phenotypic manifestations include a characteristic facies, microcephaly, hirsutism, synophrys, broad nasal bridge, congenital heart disease, genitourinary disorders, and mental retardation [5]. In approximately 60%–75% of cases, it is derived from a balanced translocation [2, 6, 7], which also presents a concomitant deletion of another chromosome; however, the clinical manifestations appear to be dominated by the dup(3q) phenotype [3, 8]. Some of the reported cases are pure partial trisomies that allowed to define a critical region in 3p26.3-q27 [2, 5, 9–11]. Here we report the clinical, cytogenetic, and biochemical characteristics of 4 patients belonging to the same family with an inherited pure duplication of 3q26.2-qter resulting from an adjacent 1 segregation from a balanced maternal t(3;13)(q26.2;p11.2). In this family, mucopolysacchariduria was also diagnosed on biochemical exams, which apparently is not related to the chromosomal abnormality or clinical manifestations.

## 2. Cases Presentation

**Case 1**

**Figure 1:**
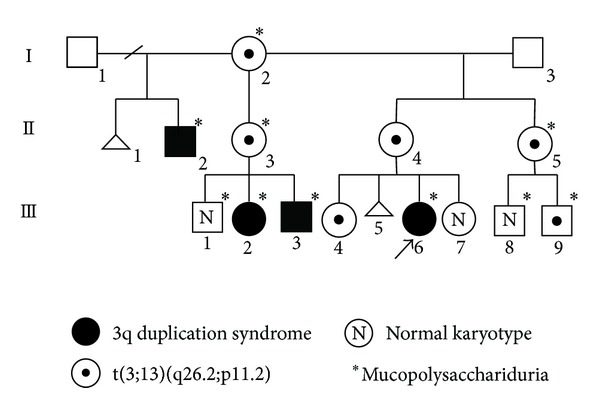
Pedigree of the family.

**Figure 2:**
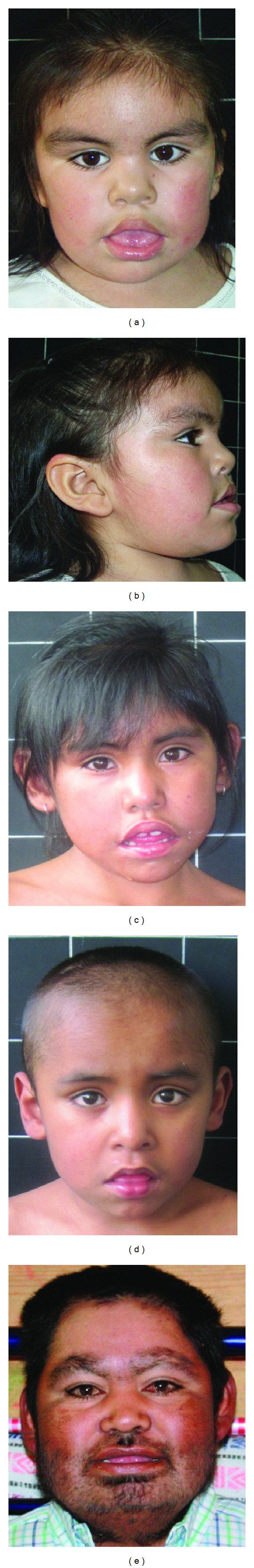
Phenotype of the four family members with the dup(3q) syndrome. (a) Frontal and (b) semilateral views of Case 1 (proband). (c) Case 2. (d) Case 3. (e) Case 4.

The proband III.6 (Figure 1) was first known as a 13 months of age female, product of a nonconsanguineous, young healthy parents. The family pedigree revealed that several members of the family presented a similar phenotype to the patient and/or had delayed psychomotor development. The pregnancy was reported with hypomotility, and she was obtained by cesarean section due to cephalopelvic disproportion. Birth weight was 4500 gr (≥p.95), height was 51 cm (p.75), and Apgar score was 8/9. She presented cyanosis on the 4th day of life, and an echocardiogram study demonstrated a normal heart. A previous metabolic screening test performed in another institution demonstrated glycosaminoglycans (GAGs) in urine. Physical examination at 13 months of age showed weight 12 kg (p.97), height 73 cm (p.25), and head circumference 46 cm (p.75). She had brachycephaly, hypertrichosis, synophrys, upward slanting palpebral fissures with long eyelashes, broad nasal bridge, anteverted nostrils, carp-shaped mouth, preauricular pits, short and wide neck, teletelia, and a 3-2-2 cm liver edge (Figures 2(a) and 2(b), Table 1). She also had developmental delay.

**Case 2**

The patient III.3 (Figure 1) is a male known at 2 months of age in our institution by cholestatic syndrome. The father was 34 years old and the mother was 24 years old at conception, both apparently healthy. He is the product of the third pregnancy, obtained at term by cesarean section due to cephalopelvic disproportion. His birth weight was 4 kg (p.90) and his height was 56 cm (p.98). The head circumference and Apgar score were unknown. At two months of age a neonatal hepatitis was confirmed by liver biopsy. At 4 years and 9 months of age he presented generalized tonic-clonic seizures, with a frequency of 1-2 per month, with difficult medical control. The physical exam was performed at 6 years of age as part of the family study and showed a global developmental delay, generalized hirsutism, normocephaly, facial dysmorphisms, upward slanting palpebral fissures, broad nasal bridge, anteverted nostrils, carp-shaped mouth, prognathism, high palate, a tendency to keep the mouth open, ears with prominent helix and preauricular pits, short neck, pectus excavatum, bilateral clinodactyly of the 5th finger, and feet brachydactyly, among other characteristics (Figure 2(c), Table 1).

**Case 3**

The patient III.2 (Figure 1) is a 7-year-old female. She was obtained at term with a weight of 3,350 g (p.50), the Apgar score is unknown, but she required advanced resuscitation maneuvers. She presents delayed psychomotor development and does not attend school. In the physical examination the weight and height were in the p.50 for her age. She is normocephalic (p.25) and has generalized hirsutism, facial dysmorphic features similar to the other members of the family, including upward slanting palpebral fissures, long eyelashes, broad nasal bridge, anteverted nostrils, high palate, carp-shaped mouth, ears in anteversion with prominent helix, preauricular pits, short neck, pectus excavatum, systolic ejection murmur II/IV, abdomen with reducible umbilical hernia of 1 cm, and bilateral clinodactyly of the 5th finger (Figure 2(d), Table 1).

**Case 4**

The patient II.2 (Figure 1) was a 35-year-old male with intellectual disability, he did not attend school and had mental retardation, his language was about 10 words, and he worked as a helper in a shop. He had generalized hirsutism, similar facial dysmorphisms, upward slanting palpebral fissures, coloboma of eyebrows, long eyelashes, broad nasal bridge, anteverted nostrils, high palate, carp-shaped mouth, multiple cavities, preauricular pits, large ears with prominent helix, short neck, and bilateral clinodactyly of the 5th finger (Figure 2(e), Table 1). He recently died in an accident at 37 years old.

## 3. Cytogenetic, Biochemical, and Molecular Analyses

### 3.1. Cytogenetic Analysis

**Figure 3:**
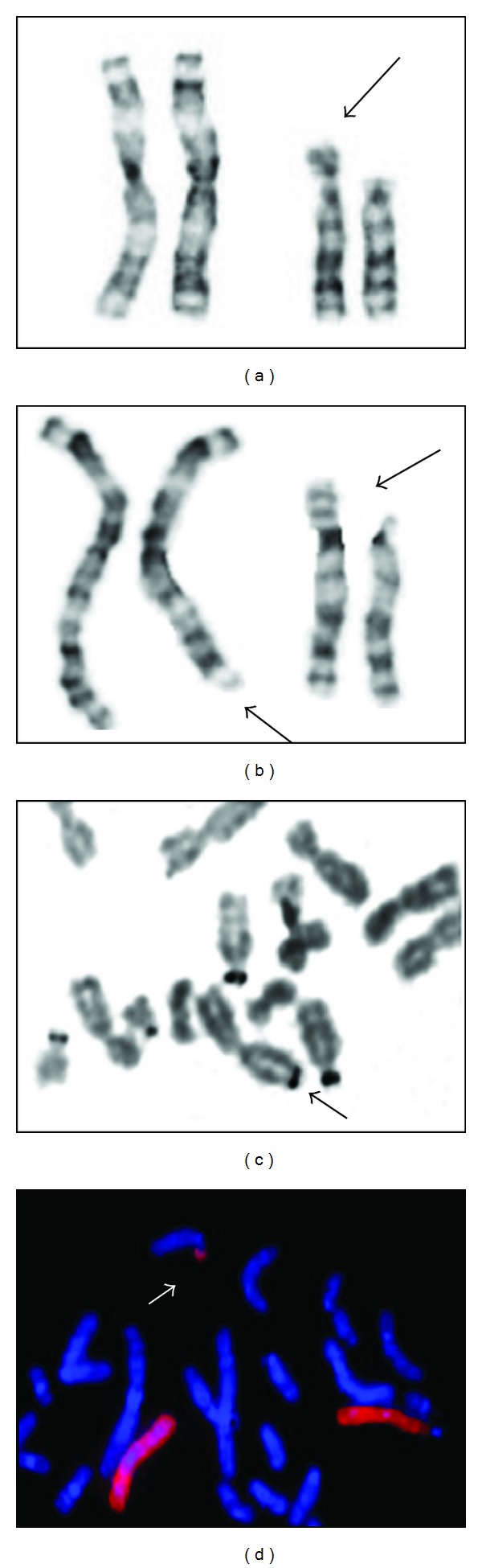
Cytogenetic analysis of blood lymphocytes. (a) Partial karyotype of the proband, the arrow shows the add(13)(q11.1). (b) GTG partial karyotype of individual II.4, the arrows show the t(3;13)(q26.2;p11.2). (c) 3q NOR positive of individual II.4 and acrocentric chromosomal aggregation. (d) FISH with WCP probe for chromosome 3 of individual II.4, arrows show der(13).

A GTG banding karyotype of the proband was initially reported as 46,XX,add(13)(p11.2). The father's karyotype was 46,XY and the mother's was 46,XX,t(3;13)(q26.2;p11.2) with a resolution of 550 bands (Figure 1, II.4), thus confirming that the additional material identified in the proband was derived from chromosome 3. A NOR banded karyotype performed in the mother demonstrated a chromosome 3 NOR positive with acrocentric association (Figure 3(c)). The molecular cytogenetic study with a whole chromosome 3 painting probe (WCP 3 Cytocell) (Cambridge, UK) (Figure 3(d)) confirmed the translocation, with a chromosome complement in the proband: 46,XX,der(13)t(3;13)(q26.2;p11.2)mat (Figure 3(a)).

The family study included GTG banding analysis in peripheral blood to the relatives, as is shown in Figure 1, and six translocation carriers (individuals I.2, II.3, II.4, II.5, III.4, and III.9) and four unbalanced individuals (II.2, III.2, III.3, and III.6), all of them with der(13)t(3;13)(q26.2;p11.2)mat, were identified.

### 3.2. Biochemical Analysis

The metabolic screening demonstrated a uronic acid/creatinine ratio consistent with mucopolysaccharidosis. The mean values of GAGs/creatinine ratio were obtained [12]. All family members who were screened for urinary GAGs excretion presented a pattern of dermatan and heparan sulfates (+) and chondroitin 4- and 6-sulfate (++), which was persistent and increases with age. *β*-glucuronidase, arylsulphatase B, and iduronate 2 sulphatase activity analysis were within the normal range. 

### 3.3. Molecular Analysis

Genomic DNA was extracted from III.6, II.4, and III.1. The exon 3 of *GUSB*, associated with Sly syndrome (MIM253220) [13], contains a pseudodeficiency allele c.454G>A (p.Asp152Asn) [14, 15]. It was amplified from these subjects, and direct automated sequencing was performed in an ABI 310 instrument (Applied Biosystems) (California, USA), and no changes were identified in any of the patients analyzed. 

## 4. Discussion

We report a family in which four members have a dup(3q) syndrome. They are derived from a familial translocation involving the short arm of chromosome 13. The absence of the nucleolar organizer region (NOR) does not impact on the phenotype as the NOR of the other acrocentric chromosomes compensates their function, and therefore these patients correspond to a pure 3q duplication syndrome, which will help to further delineate this syndrome (Table 1).

To our knowledge, there is only a case reported with a dup(3q) syndrome resulting from a balanced translocation involving chromosomes 3 and 13, and it was diagnosed prenatally. Amniocentesis was performed yielding a karyotype 46,XY,der(13)t(3;13)(q12;q11.1)mat, and a therapeutic abortion was performed [16]. The referred case involved a more proximal chromosome break point on chromosome 3 and apparently had clinical data similar to the described for a dup(3q) syndrome. It is remarkable that there are other reports of pure dup(3q) syndrome involving a rearrangement with the short arm of an acrocentric chromosome [7, 17, 18]. The family described, as in the case reported by Falek et al. [17], involves an extended family with various balanced carriers and affected individuals. Interestingly, the origin of the derived chromosome is maternal in most of the cases.

When comparing the phenotype of our patients with the clinical manifestations of the few cases of pure dup(3q) syndrome previously reported (Table 1), they share the main characteristics of the syndrome. For example, most of the facial phenotype described for the critical region of the dup(3q) syndrome is present in our patients; they also have hirsutism, dysmorphic ears, and short neck, but they do not show microretrognathia. Taking together this information, our cases contribute to confirm that the critical region for facial characteristics is 3q26.3-q27; however, the implication of this region for other characteristics is not as clear; for example, the only limb abnormality present in our patients was clinodactyly (Table 1), and there are some characteristics that although they are not present in all the cases have a high frequency of presentation, such as growth retardation and microcephaly.

Regarding the growth retardation it has been described in 63% of the cases; however, none of our patients had this characteristic, and it was described in the patients reported by Falek et al. [17], which share the same chromosome 3q duplicated region. The presence of microcephaly has been described in 60% of the cases, but none of our patients had it; this characteristic is present more frequently with a more proximal duplicated region than in the cases with only an abnormality in 3q26 [4, 6, 9, 19, 20]. It is interesting to note that patients with microduplication 3q29 also had microcephaly in a high proportion (50%). This assumption is not an absolute one as there is another group of patients who in addition to the critical region for the dup(3q) syndrome has a 3q29 duplication and does not show this characteristic [21–23]. We may consider that microcephaly as is the case with growth retardation involves complex structures and characteristics, and therefore several genes must be implicated.

Some other anomalies described in dup(3q) syndrome are seizures [6, 9, 17], but only one of our patients presented this, as what happened for the cardiopathy (Table 1). Once again, some patients with microduplication of 3q29 also had cardiopathy (31%), and therefore these complex characteristics and their variation in the clinical presentation may be related to multifactorial inheritance, and probably several predisposition genes may be located along 3q. The microduplication 3q29 syndrome is a different entity and does not present the classical phenotype of dup(3q); several patients have mental retardation, microcephaly, and cardiopathy, and some patients with dup(3q) syndrome have also duplicated 3q29, so this region could also contribute to the phenotype. The survival of these patients is related to the severity of the malformations.  Case 4 (II.2) described here reached adulthood probably due to the absence of congenital heart disease; this may be the first pure dup(3q) classical syndrome reported patient with survival to adulthood. 

Faas et al. [5] proposed that there should be genes within the critical region involved in the development of the typical dysmorphia and the congenital abnormalities of the dup(3q) syndrome. One of the genes that may explain the clinical manifestations is *NLGN1* (MIM*600569), which encodes neuroglia 1, involved in the synaptogenesis of the central nervous system, and its alteration might explain mental retardation in dup(3q) syndrome patients [8]. It has been proposed that other genes located in 3q26.1–q26.3, such as *GHSR* (MIM*601898), *CLDN11* (MIM*601326), *PLD1* (MIM*602382), and *ECT2* (MIM*602382), are related to the development of the neural system, and in particular *GHSR* is also associated with short stature [20]; therefore some authors consider that dup(3q) syndrome is a contiguous gene syndrome in which the altered gene dosage explains the various characteristics of the phenotypes displayed in the patients [4].

In the study of this family, we identified six balanced translocation carriers and four unbalanced patients, and genetic counseling was offered to the family. The empirical risk of recurrence for children of carriers of a translocation t(3;13) is calculated at 16.31% [24, 25], and it is probably lower than the theoretical risk because altered gene dosage during embryogenesis causes abortions. Our four patients rearrangements resulted from an adjacent 1 segregation, interestingly with the exception of one male; all the balanced carriers are females (Figure 1).

An interesting fact in this family is that they also present urine GAGs excretion, initially indicating the possibility of a mucopolysaccharidosis disease. We found no association of urinary excretion and the carrier translocation status or partial trisomy 3q. This led us to conclude that the mucopolysacchariduria is not associated with the chromosomal imbalance or the allele pseudodeficiency of *GUSB* (Figure 1).

## 5. Conclusion

This case illustrates a pure typical partial trisomy 3q syndrome derived from a maternal balanced translocation t(3;13)(q26.2;p11.2) because the chromosomal rearrangement involves the short arm of an acrocentric chromosome; the phenotype corresponds to a pure trisomy 3q26.2-qter. The recurrence risk for children of carriers of the translocation is calculated at 16.31%. The report of this family is important because there are few cases of pure duplication 3q syndrome reported, and the four cases described here contribute to define the phenotype associated with the syndrome. Furthermore, we confirmed that the survival until adulthood is possible. This report also identified the presence of GAGs in urine in this family, probably not related to the chromosomal abnormality or the phenotype.

## Figures and Tables

**Table 1:** 

	[17] 4 cases^a^	[9] 2 cases^b^	[6]	[10]	[2]	[19] 2 cases	[5]	[8]	[11]	[4]	[20]	Our study 4 cases		Microduplication 3q29 [21–23, 26] 13 cases
3q duplicated region origin	q26.2-qter mat/pat familial	q25–q29 *de novo *	q23–q27 *de novo *	q21–q26^c^ *de novo *	q25–q28 *de novo *	q25.1–q26.2 mat	q26.3-qter *de novo *	q24–q26.31 *de novo *	q27-qter^d^ *de novo *	q22.2–q29 *de novo *	q24–q28^e^ pat	q26.2-qter mat familial	Frequency of clinical manifestations	q29
Cytogenetic analysis	GTG/FISH/CGH^f^	GTG	GTG	GTG/QFQ/RFA	GTG/SB	GTG/FISH/CGH	GTG/CGH FISH/SKY	GTG/QFQ/RBA/FISH	GTG/FISH SNPa	GTG/FISH SNPa	GTG/FISH/SKY/SNPa	GTG/NOR/FISH		SNPa/aCGH/GTG
Oldest age recorded gender	9 m–14 y 3 F/1 M	9 y 2 m/8 y F/M	26 m F	9 m M	11 m F	10 y 6 m/ND M/F	11 m M	15 m M	4 y 8 m F	42 m M	32 m M	3 y 3 m–35 y 3 F/2 M		6 m–56 y
Developmental delay/mental retardation	4/4	2/2	1/1	1/1	1/1	2/2	1/1	1/1	1/1	1/1	1/1	4/4	20/20 100%	11/13 85%
Growth retardation	4/4	2/2	1/1	1/1	ND	0/2	1/1	1/1	0/1	1/1	1/1	0/4	12/19 63%	ND
Facial features														
Microcephaly	4/4	2/2	1/1	0/1	0/1	2/2	0/1	1/1	0/1	1/1	1/1	0/4	12/20 60%	6/12^f^ 50%
Low hairline	1/3	2/2	1/1	1/1	ND	ND	1/1	ND	0/1	0/1	0/1	4/4	10/17 58%	1/13 8%
Hirsutism	4/4	2/2	1/1	0/1	1/1	0/2	0/1	0/1	0/1	1/1	0/1	4/4	13/20 65%	0/13 0%
Synophrys	4/4	2/2	1/1	0/1	1/1	0/2	0/1	0/1	0/1	1/1	0/1	4/4	13/20 65%	0/13 0%
Bushy eyebrows	4/4	2/2	1/1	0/1	1/1	0/2	1/1	0/1	0/1	1/1	0/1	4/4	14/20 70%	0/13 0%
Long eyelashes	4/4	2/2	1/1	0/1	1/1	0/2	1/1	0/1	0/1	1/1	0/1	3/4	13/20 65%	0/13 0%
Wide nasal bridge	4/4	0/2	1/1	1/1	1/1	1/2	1/1	1/1	1/1	1/1	1/1	4/4	18/19 94%	4/13 31%
Anteverted nostrils	4/4	2/2	1/1	1/1	ND	2/2	1/1	1/1	0/1	1/1	1/1	4/4	18/19 94%	0/13 0%
Downturned corners of the mouth	4/4	1/2	1/1	1/1	ND	1/2	1/1	1/1	1/1	1/1	1/1	4/4	17/19 89%	3/13 23%
High/cleft palate	0/4	0/2	1/1	1/1	1/1	0/2	0/1	0/1	0/1	0/1	1/1	0/4	4/20 20%	4/13 31%
Micro/ retrognathia	3/4	2/2	0/1	1/1	1/1	0/2	1/1	1/1	0/1	1/1	0/1	0/4	10/20 50%	0/13 0%
Dysmorphic ears	3/4	0/2	0/1	1/1	ND	0/2	1/1	1/1	0/1	0/1	1/1	4/4	11/19 57%	0/13 0%
Limb abnormalities														
Brachydactyly	4/4	2/2	0/1	0/1	1/1	2/2	1/1	1/1	1/1	1/1	0/1	1/4	1420 70%	0/13 0%
Clinodactyly	3/4	2/2	1/1	1/1	1/1	2/2	0/1	0/1	0/1	1/1	0/1	4/4	14/20 70%	0/13 0%
Other limbs abnormalities	0/4	0/2	0/1	0/1	0/1	2/2	1/1	1/1	1/1	1/1	1/1	0/4	7/20 35%	4/13 31%
Other														
Short neck	3/4	2/2	0/1	1/1	ND	2/2	1/1	1/1	0/1	1/1	0/1	4/4	15/19 78%	0/13 0%
Cardiopathy	2/4	ND	1/1	1/1	1/1	2/2	1/1	1/1	0/1	0/1	1/1	1/4	11/19 57%	4/13 31%
Seizures disorders	0/4	2/2	ND	ND	ND	0/2	0/1	ND	0/1	1/1	0/1	1/4	4/16 25%	0/13 0%

F: female; M: male; y: years; m: months; ND: no determined; mat: maternal; pat: paternal; GTG: G-bands by trypsin using Giemsa; RBA: R-bands by BrdU using acridine orange; RFA: R-bands by fluorescence using acridine orange; QFQ: Q-bands by fluorescence using quinacrine; SB: southern blot; FISH: fluorescence in situ hybridization; CGH: comparative genomic hybridization on metaphase; aCGH: array comparative genomic hybridization; SNPa: single nucleotide polymorphism array. ^a^One case clinically affected, died prior to chromosome analysis, initial karyotype without banding. ^b^One case had also a monosomy of 3p27-pter. ^c^Probably with del(3)(q27q29); ^d^the karyotype was 46,XX,der(4)t(3;4)(q27;p16); the 4p deletion is of less than 500 kb, so it is considered a 3q27-qter pure trisomy. ^e^Karyotype: 46,XYins(6;3)(q21;q24q28). ^f^The karyotype was done by Rizzu and Baldini [19]; the final karyotype was 46,XY,der(22)t(3;22)(q26;p11). ^f^1 case reported by Balif et al. [21] had macrocephaly.
